# Supplementary material for: Clinical and Virological Features of Patients Hospitalized with Different Types of COVID-19 Vaccination in Mexico City
Source: Vaccines (Basel). 2022 Jul 26;10(8):1181. doi: 10.3390/vaccines10081181 (PMC9330015; doi:10.3390/vaccines10081181)
Supplement: Supplementary file 1 [file vaccines-10-01181-s001.zip › vaccines-1788105-supplementary.pdf]

Clinical and virological features of patients hospitalized with different types of COVID-19 vaccination in Mexico City

SUPPLEMENTARY MATERIAL

Supplementary Information S1. Specifics for patient vaccination categories.

Categories:

1) Unvaccinated: patients who had never received a SARS-CoV-2 vaccine.

2) Partially vaccinated: patients who had not yet completed the standard vaccination schedule (depending on the vaccine) or had received the last dose less than 14 days before symptom onset.

3) Fully vaccinated: patients who had already completed the vaccination schedule and with more than 14 days after the last dose.

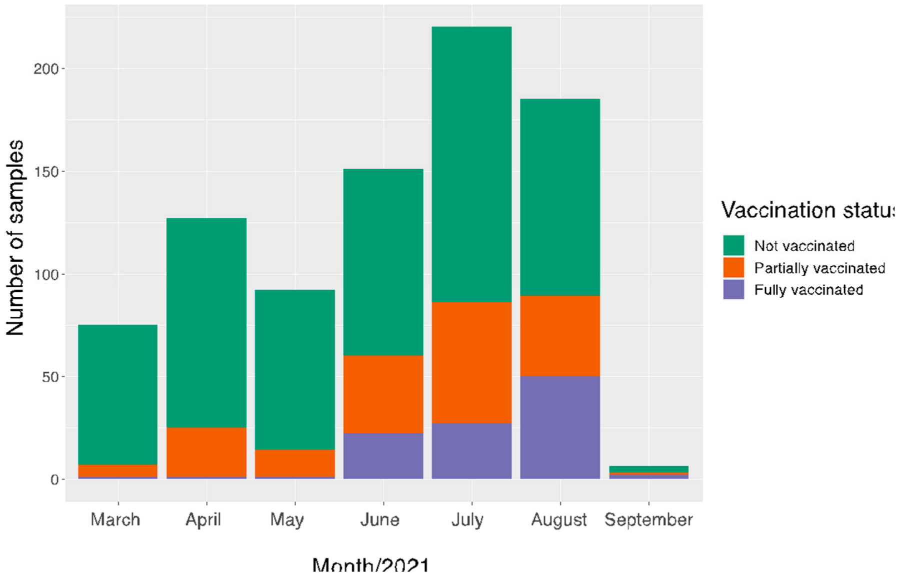

Supplementary Figure S1. Vaccination status of all patients studied from March to September 2021.

Supplementary Table S1. COVID-19 classification among patients with different vaccination statuses.

	**<30 years old**(N=85)				**31-60 years old**(N=599)				**>61 years old**(N=278)			
	**NV**	**PV**	**FV**	*p*	**NV**	**PV**	**FV**	*p*	**NV**	**PV**	**FV**	*p*
**Mild**	13(19%)	3(75%)	14 (100%)	***<0.001***	25(6.1%)	22(14.1%)	17(47.2%)	***<0.001***	1(0.72%)	6(11.7%)	8(8.8%)	***0.002***
**Severe**	23(34.3%)	0	0	*ns*	137(33.6%)	55(35.2%)	8(22.2%)	*ns*	34(24.8%)	19(37.2%)	31(34%)	*ns*
**Critical**	31(46.2%)	1(25%)	0	*ns*	245(60.1%)	79(50.6%)	11(30.5%)	***<0.001***	102(74.4%)	26(50.9%)	50(55%)	***0.001***
Total	67	4	14		407	156	36		137	51	90	

NV: not vaccinated
PV: partially vaccinated
FV: fully vaccinated
p values were obtained from a Chi-square or Fisher's exact test. ns: non-significant. Statistically significant values are in bold.

Supplementary Figure S2. Distribution of ages and severity of the disease in fully vaccinated patients with different vaccine strategies.

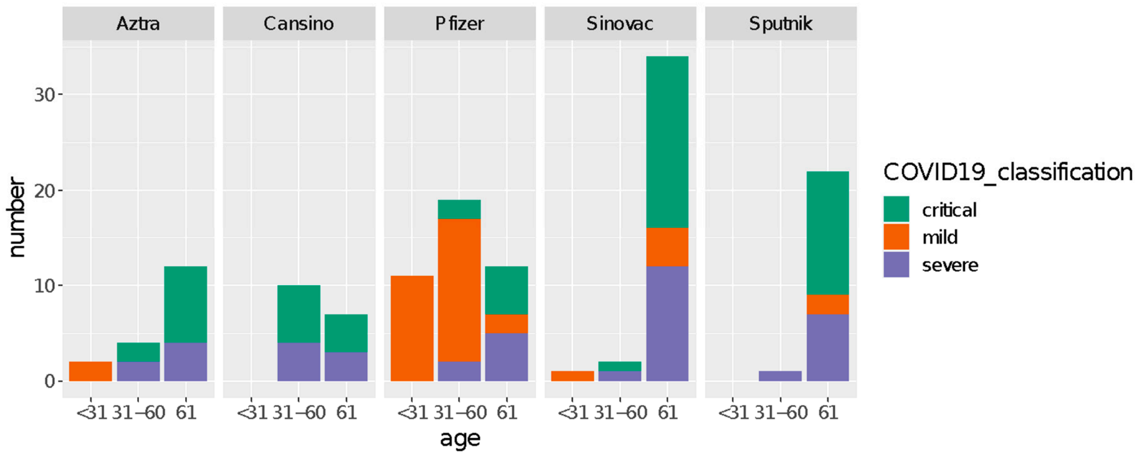

Supplementary Table S2. Statistical differences in the severity of the disease between patients with different vaccine types and age groups

	Classification	**Pfizer** (N=12)	**AstraZeneca** (N=12)	**Sinovac** (N=35)	**Sputnik** (N=22)	**Cansino** (N=7)
**>61** (N=88)	Mild	2(16.6%)	0	4 (11.4%)	2 (9%)	0
	Severe	5(41.6%)	4 (33.3%)	12 (34.2%)	7 (31.8%)	3 (42.8%)
	Critical	5(41.6%)	8 (66.6%)	18 (51.4%)	13 (59%)	4 (57.1%)
	NA	0	0	1 (2.8)	0	0
	P value	*ns*	***0.002***	***0.001***	***0.002***	*ns*

*We excluded two patients from the table: one vaccinated with Moderna and the other vaccinated with J&J.
p values were obtained from a Chi-square or Fisher's exact test.
ns: non-significant, **na:** comparison not available.
Statistically significant values are in bold.

Supplementary Table S3. Statistical differences of the O_2 requirement between patients with different vaccine types and age groups.

	Classification	**Pfizer** (N=42)	**AstraZeneca** (N=19)	**Sinovac** (N=40)	**Sputnik** (N=23)	**Cansino** (N=17)	**P** ***value***
**All** (N=141)*	Nasal cannula	4 (9.5%)	1 (5.26%)	2 (5%)	3 (13%)	2 (11.7%)	*ns*
	HFNC	3 (7.14%)	3 (15.78%)	12 (30%)	4 (17.3%)	5 (29.4%)	***<0.001***
	Reservoir OM	0	0	0	1 (4.34%)	0	*na*
	NIMV	0	0	1 (2.5%)	0	0	*na*
	MV	7 (16.6%)	10 (52.6%)	19 (47.5%)	13 (56.5%)	10 (58.8%)	***0.02***
	None	0	0	0	0	0	*na*
	NA	28 (66.6%)	5 (26.3%)	6 (15%)	2 (8.6%)		***<0.001***
	P value	*ns*	***0.002***	***<0.001***	***<0.001***	*Ns*	*na*
**>61** (N=88)	Nasal cannula	2 (16.6%)	1 (8.3%)	2 (5.7%)	2 (9%)	0	***0.01***
	HFNC	3 (25%)	3 (25%)	10 (28.5%)	4 (18.1%)	3 (42.8%)	***0.01***
	Reservoir OM	0	0	0	1 (4.5%)	0	*na*
	NIMV	0	0	1 (2.8%)	0	0	*na*
	MV	5 (41.6%)	8 (66.6%)	17 (48.5%)	13 (59%)	4 (57.1%)	*ns*
	None	0	0	0	0	0	*na*
	NA	2 (16.6%)	0	0	0	0	*na*
	P value	*ns*	***0.01***	***<0.001***	***<0.001***	*ns*	*na*

*We excluded two patients from the table: one vaccinated with Moderna and the other vaccinated with J&J.
p values were obtained from a Chi-square or Fisher's exact test.
ns: non-significant, **na:** comparison not available.
Statistically significant values are in bold.
HFNC: high-flow nasal cannula.
Reservoir OM: reservoir oxygen mask.
NIMV: non-invasive mechanical ventilation.

Supplementary Table S4. Odds ratio and estimates of the two generalized linear models (GLMs).

	Estimate	Std. Error	Z value	Odds ratio	95% CI	Pr(>z)
Model 1						
**(Intercept)**	-1.5617	0.1375	-11.35	-	0.15-0.273	<0.001***
**Partially vaccinated**	-0.1009	0.2223	-0.454	0.90	0.57-1.38	***0.64***
**Fully vaccinated**	-1.3948	0.3335	-4.182	0.25	0.12-0.46	2.89e-05***
**Age > 61**	1.2569	0.2030	6.192	3.51	2.31-5.2	5.9e-10***
**Diabetes**	-0.1981	0.2171	-0.912	0.82	0.52-1.2	*0.36*
**Immune disease**	1.1385	0.5097	2.234	3.12	1.09-8.3	0.02*
**Sex (Women)**	-0.1212	0.1884	-0.643	0.89	0.60-1.27	*0.52*
Model 2						
**(Intercept)**	-1.6283	0.2671	-6.096	-	0.11-0.327	1.09e-09***
**Platelets < 175**	0.3979	0.2082	1.911	1.49	0.98-2.22	0.05*
**BUN > 39**	-0.311	0.2422	-1.284	0.73	0.45-1.18	***0.19***
**D-Dimer > 1.8**	0.3693	0.1963	1.882	1.45	0.98-2.11	0.05*
**LDH > 600**	0.6344	0.1860	3.9	1.89	1.28-2.75	0.001**

Supplementary Table S5. Statistical differences in lineages' distribution in the samples sequenced of Mexico, State of Mexico, Mexico City, and INER.

SARS-CoV-2 lineage	Mexico	State of Mexico	Mexico City	INER	*P* value
**Delta**, n (%)	15,301 (51.6%)	1,377 (51.2%)	3248 (50.6%)	265 (69%)	***<0.001***
**Alpha**, n (%)	1,787 (6.03%)	178 (6.6%)	291 (4.5%)	9 (2.3%)	***<0.001***
**Gamma**, n (%)	2,749 (9.2%)	144 (5.36%)	325 (5%)	28 (7.2%)	***<0.001***
**Mu**, n (%)	434 (1.4%)	58 (2.1%)	152 (2.36%)	14 (3.6%)	***<0.001***
**B.1.1.519**, n (%)	6,265 (21.1%)	711 (26.4)	1,986 (30.9%)	57 (14.8%)	***<0.001***
**Other lineages**, n (%)	3,085 (10.4%)	217 (8%)	412 (6.4%)	11 (2.8%)	***<0.001***
Total sequences	29,621	2685	6414	384	

Data from Mexico, State of Mexico, and Mexico City were taken from gisaid.com.
P values were obtained from a Chi-square analysis. Statistically significant values are in bold.

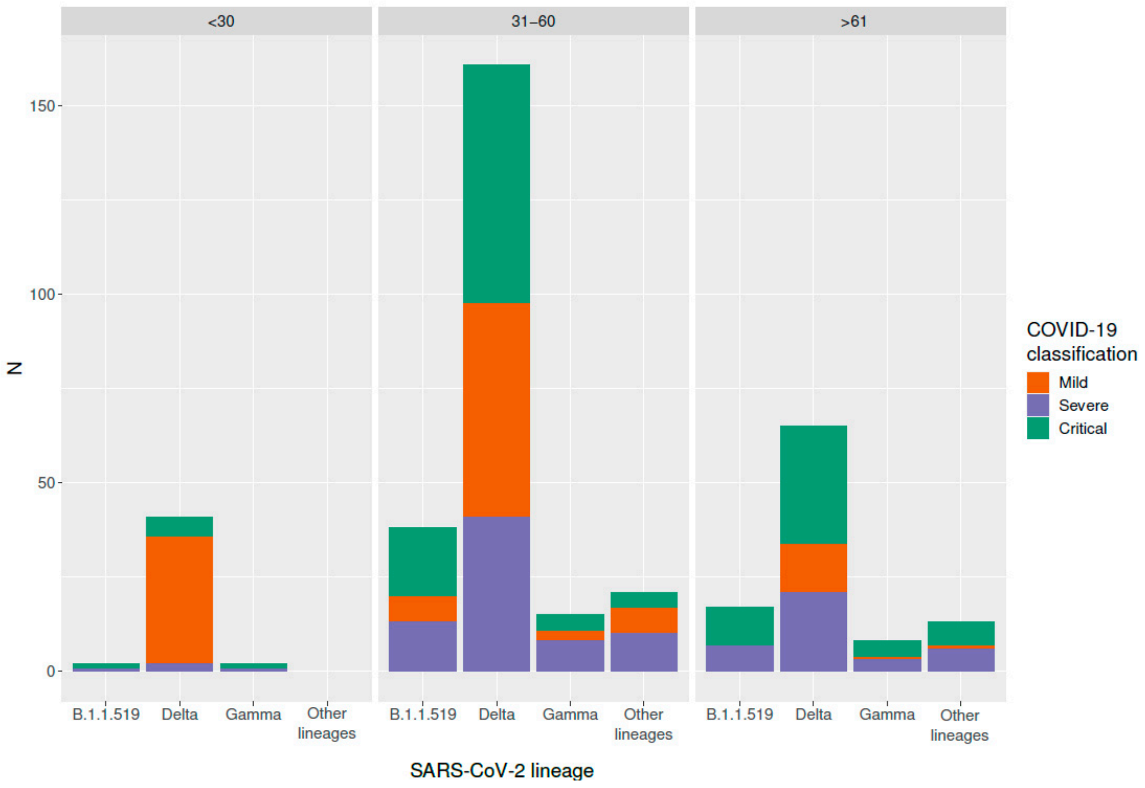

	**<30** (N=44)				**31-60** (N=237)					**>61** (N=103)				
Status/variant	**Delta** (N=40)	**Gamma** (N=2)	**B.1.1.519** (N=2)	*p*	**Delta** (N=160)	**Gamma** (N=18)	**B.1.1.519** (N=38)	**Other** (N=21)	*p*	**Delta** (N=65)	**Gamma** (N=8)	**B.1.1.519** (N=17)	**Other** (N=13)	*p*
**Mild** (N= 121)	33(82.5%)	0	0	*na*	56(35%)	3(16.6%)	7(18.4%)	7(33.3%)	*ns*	13(20%)	1(12.5%)	0	1(7.6%)	*ns*
**Severe** (N=113)	2(5%)	1(50%)	1(50%)	***0.03***	41(25.6%)	8(44.4%)	13(34.2%)	10(47.6%)	***0.05***	21(32.3%)	3(37.5%)	7(41.1%)	6(46.1%)	*ns*
**Critical** (N=150)	5(12.5%)	1(50%)	1(50%)	*ns*	63(39.3%)	7(38.8%)	18(47.3%)	4(19%)	*ns*	31(47.6%)	4(50%)	10(58.8%)	6(46.1%)	*ns*
P	***<0.001***	*ns*	*ns*		***0.02***	*ns*	***0.02***	*ns*		***0.003***	*ns*	***0.003***	*ns*	

p values were obtained from a Chi-square or Fisher's exact test.
na: comparison not available
ns: non-significant

Supplementary Figure S3. COVID-19 classification among patients infected with different lineages. A. Bar plot of patients infected with different lineages and their hospitalized status, controlled by age. B. Statistically significant comparisons between groups, and p values were obtained from a Chi-square test.

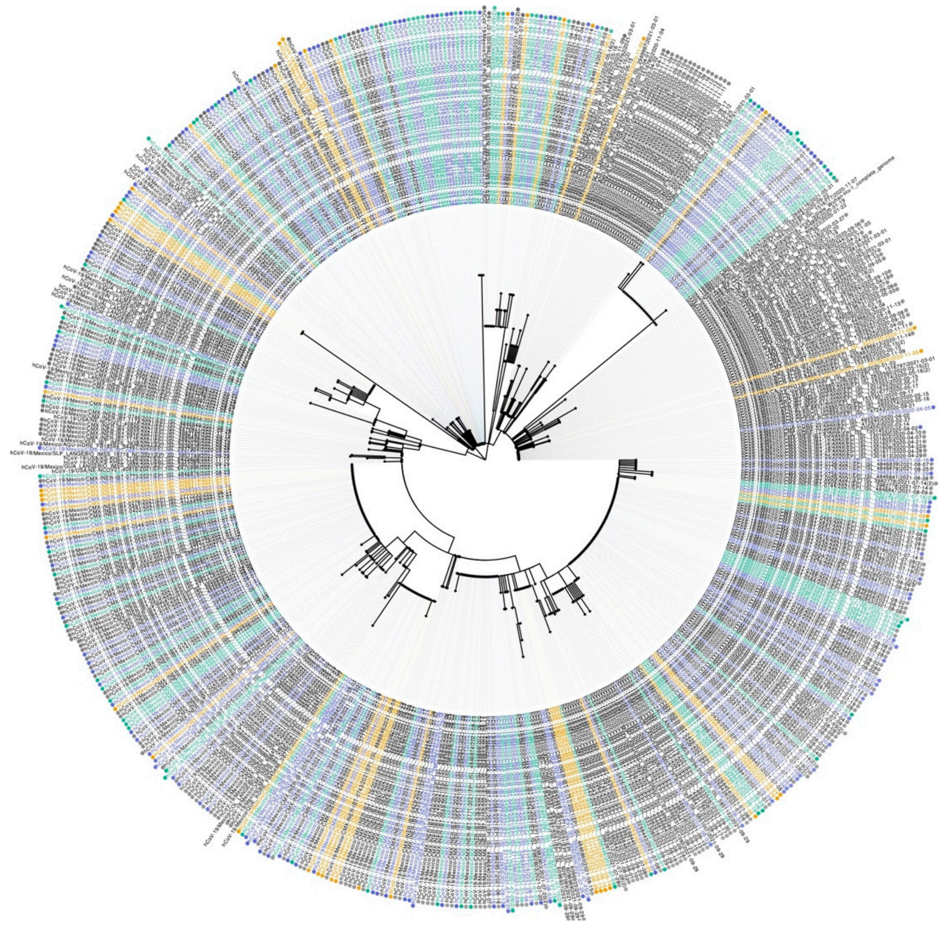

Supplementary Figure S4. Phylogeny. Maximum-likelihood (ML) phylogenetic tree for the Spike whole sequence. ML tree from 541 sequences registered in GISAID was produced with 1,000 bootstrap replicates. The sequences obtained in this study are included. Branches are colored according to vaccination status—orange: unvaccinated, green: partially vaccinated, and purple: fully vaccinated patients.
